# The Nuclear Remodeling Induced by Helicobacter Cytolethal Distending Toxin Involves MAFB Oncoprotein

**DOI:** 10.3390/toxins12030174

**Published:** 2020-03-12

**Authors:** Christelle Péré-Védrenne, Wencan He, Lamia Azzi-Martin, Valérie Prouzet-Mauléon, Alice Buissonnière, Bruno Cardinaud, Philippe Lehours, Francis Mégraud, Christophe F. Grosset, Armelle Ménard

**Affiliations:** 1 Université de Bordeaux, INSERM—Institut National de la Santé et de la Recherche Médicale, BaRITOn—Bordeaux Research in Translational Oncology, UMR1053, 33076 Bordeaux, France; c.pere.vedrenne@gmail.com (C.P.-V.); wencsilence@126.com (W.H.); lamia.azzi-martin@u-bordeaux.fr (L.A.-M.); Alice.fly@hotmail.fr (A.B.); philippe.lehours@u-bordeaux.fr (P.L.); francis.megraud@chu-bordeaux.fr (F.M.); 2 Université de Bordeaux, TBMCore, CRISP’edit, TBMcore CNRS-Centre National de la Recherche Scientifique UMS3427/INSERM—Institut National de la Santé et de la Recherche Médicale US005, 33076 Bordeaux, France; Valerie.Prouzet-Mauleon@u-bordeaux.fr; 3 Université de Bordeaux, INSERM—Institut National de la Santé et de la Recherche Médicale, ACTION, U1218, Institut Bergonié, 33076 Bordeaux, France; Bruno.Cardinaud@u-bordeaux.fr; 4 Bordeaux INP, ENSTBB, F-33000 Bordeaux, France; 5 CHU Pellegrin, National Reference Center for Campylobacters and Helicobacters, 33076 Bordeaux, France; 6 Université de Bordeaux, INSERM—Institut National de la Santé et de la Recherche Médicale, BMGIC—Biotherapy of Genetic Diseases, Inflammatory Disorders and Cancer, U1035, miRCaDe Team, 33076 Bordeaux, France; christophe.grosset@u-bordeaux.fr

**Keywords:** *Helicobacter hepaticus*, *Helicobacter pullorum*, CdtB subunit, cytolethal distending toxin, MAFB oncoprotein

## Abstract

Enterohepatic Helicobacters, such as *Helicobacter hepaticus* and *Helicobacter pullorum,* are associated with several intestinal and hepatic diseases. Their main virulence factor is the cytolethal distending toxin (CDT). In the present study, whole genome microarray-based identification of differentially expressed genes was performed in vitro in HT-29 intestinal cells while following the ectopic expression of the active CdtB subunit of *H. hepaticus* CDT. A CdtB-dependent upregulation of the V-maf musculoaponeurotic fibrosarcoma oncogene homolog B (*MAFB*) gene encoding the MAFB oncoprotein was found, as well as the CdtB-dependent regulation of several MAFB target genes. The transduction and coculture experiments confirmed *MAFB* mRNA and protein induction in response to CDT and its CdtB subunit in intestinal and hepatic cell lines. An analysis of MAFB protein subcellular localization revealed a strong nuclear and perinuclear localization in the CdtB-distended nuclei in intestinal and hepatic cells. MAFB was also detected at the cell periphery of the CdtB-induced lamellipodia in some cells. The silencing of MAFB changed the cellular response to CDT with the formation of narrower lamellipodia, a reduction of the increase in nucleus size, and the formation of less γH2AX foci, the biomarker for DNA double-strand breaks. Taken together, these data show that the CDT of enterohepatic Helicobacters modulates the expression of the MAFB oncoprotein, which is translocated in the nucleus and is associated with the remodeling of the nuclei and actin cytoskeleton.

## 1. Introduction

The involvement of chronic bacterial infections in carcinogenesis was first proven with *Helicobacter pylori* infection in the development of two different gastric cancers in humans. Enterohepatic *Helicobacter* species are also associated with several intestinal and/or hepatic diseases (as reviewed in [1]). Most of them possess the cytolethal distending toxin (CDT). *Helicobacter hepaticus* CDT causes chronic inflammatory lesions in mice, leading to hepatocarcinoma in older animals [2]. *H. hepaticus* CDT also promotes colitis and intestinal carcinogenesis in susceptible mice [3]. CDT is widely distributed among gram-negative bacteria. It consists of three protein subunits, CdtA, CdtB, and CdtC, with CdtB being the active subunit. CDT toxicity is dependent on CdtB internalization into the nucleus of the host cell that requires CdtA and CdtC subunits (as reviewed in [4]). Thus, directly expressing CdtB into the cells is a complementary method for coculturing experiments to study the effects specifically related to the toxin. In light of that, we previously validated a two-way original system composed of (1) coculture experiments with Helicobacter strains and (2) a lentivirus-based system for directly expressing the CdtB subunit into the cells [5,6]. Coculture experiments with Helicobacter strains and their corresponding ΔCDT isogenic mutant strains allowed for an examination of non-CDT bacterial factors in the effects that were observed while lentivirus-based expression of the CdtB and its corresponding mutated CdtB (CdtB-H265L) lacking catalytic activity [7] enabled an analysis of the effects specifically related to the CdtB [6,8].

We performed a whole genome microarray-based identification of differentially expressed genes in response to the CdtB subunit of *H. hepaticus* in transduced intestinal epithelial cells [6]. These chip analyses showed a CdtB-dependent upregulation of six members of the Activator Protein-1 (AP-1) superfamily whose V-maf musculoaponeurotic fibrosarcoma oncogene homolog B (*MAFB)* oncogene encodes the MAFB transcription factor. AP-1 is involved in processes, including differentiation, proliferation, and apoptosis. AP-1 is also implicated in the control of various cancer cells [9], including those that are involved in colorectal cancer [9,10]. The AP-1 transcription factor family is comprised of four sub-families, including members from the JUN (JUN/c-JUN, JUNB, and JUND), FOS (FOS/c-FOS, FOSB, FOS-L1/FRA-1, and FOS-L2/FRA-2), ATF (ATF-2, ATF-3, ATF-4, ATF-5, ATF-6, ATF-6B, ATF-7, BATF, BATF-2, BATF-3, and JDP2), and MAF protein families. The MAF family encompasses three small (MAFF, MAFG, and MAFK) and four large (MAFA, MAFB, MAF, NRL) leucine-zipper (bZip) proteins [9]. MAFB is a large bZip transcription factor that is characterized by the presence of an acidic N-terminal transactivation domain. MAFB plays an important role in the regulation of lineage-specific hematopoiesis. It acts as a key regulator in mammalian gene regulation and cell differentiation. MAFB is a bona fide oncogene in human cancers that is able to transactivate and transform primary cells [9,11]. Some MAFB-induced phenotypes (i.e., actin cytoskeleton reorganization, lamellipodia formation, and proliferation/cell cycle arrest [12]) are reminiscent of those that are induced by the CDT [5,8,13,14]. The effects of the CDT of enterohepatic *Helicobacter* species on *MAFB* gene regulation were thus evaluated on human intestinal and hepatic epithelial cell lines using the validated two-way system described above [5,6], as these bacilli colonize the intestine and the liver. MAFB protein expression was also investigated to determine its cellular expression and localization. MAFB silencing was then performed using the CRISPR-Cas9 technology and the remodeling of the actin cytoskeleton was evaluated.

## 2. Results

### 2.1. The MAFB Oncogene Is Upregulated in Response to the CdtB of Helicobacter

**Figure 1:**
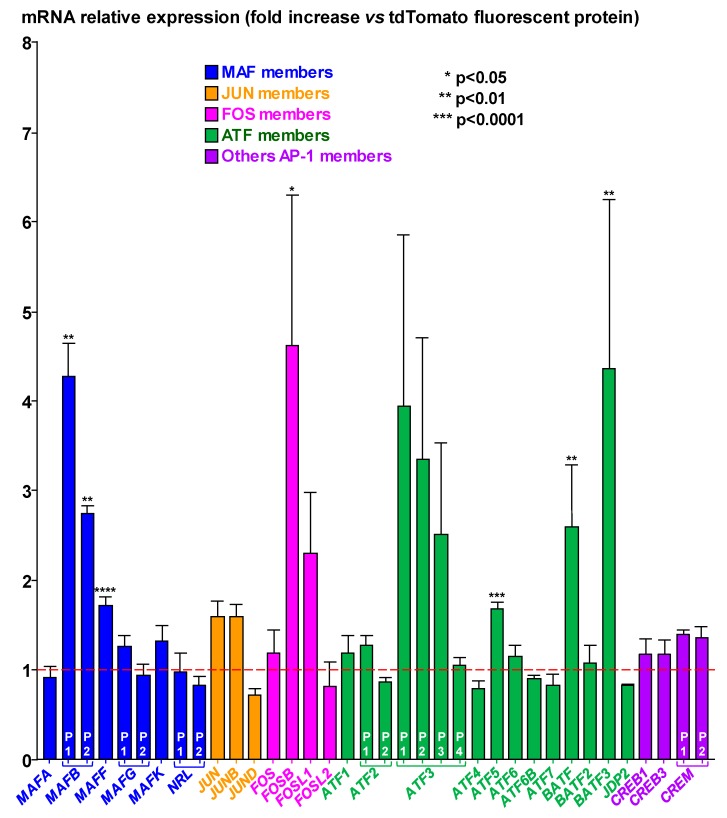
Microarray-based identification of differentially expressed Activator Protein-1 family members in response to *Helicobacter hepaticus* CdtB in intestinal cells. The expression of genes was determined in HT-29 intestinal cells using the Human GE 4x44K v2 Microarray Kit (Agilent Technologies) after a 72 h transduction with lentiviral particles expressing the CdtB of *H. hepaticus* strain 3B1 versus the tdTomato fluorescent protein (TFP). The relative expression of genes in response to CdtB is reported as a fold change versus the value for cells cultured with lentiviral particles expressing the TFP. The results are presented as the mean of four independent transduction experiments. The red discontinuous line shows the basal rate in cells expressing TFP. Statistical and bioinformatic analyses were performed as previously reported [6]. Asterisks denote significant results. P1, P2, P3, and P4 represent the probe names of the corresponding gene used for mRNA quantification. The data presented for *MAFF* (P103110), *ATF5* (P119337) and *JDP2* (P117582) are the results of 40 replicates as 10 probes for each mRNA were included on the Microarray Kit. Details are presented in Table S1 (name and sequence of the probes, the corresponding gene name, the genbank accession number, the locus and the transcript variant). The *MAF*/*c-MAF* gene is not reported as this gene was slightly expressed in HT-29 cell line and hybridization results were not conclusive. Abbreviations: AP-1, Activator Protein-1, ATF, Activating Transcription Factor; BATF, Basic Leucine Zipper ATF-Like Transcription Factor; CAMP, Responsive Element Modulator; CREB, CAMP Responsive Element Binding Protein; FOS-L1/FRA-1, FOS Like 1; FOS-L2/FRA-2, FOS Like 2; JDP2, JUN Dimerization Protein 2; NRL, Neural Retina Leucine Zipper; TFP, tdTomato fluorescent protein.

During transduction experiments with lentivirus particles expressing the CdtB subunit of *H. hepaticus*, the previously reported cytopathogenic effects associated with CdtB [5,8,13,14] were observed, including actin cytoskeleton remodeling associated with cellular distension, enlarged cells with distended or multinucleated nuclei, and the formation of cortical actin-rich large lamellipodia [5,8], therefore validating the lentiviral approach. The global expression of human genes was quantified in transduced epithelial intestinal HT-29 cells using whole genome microarrays following ectopic expression of the CdtB of *H. hepaticus* versus the control tdTomato fluorescent protein (TFP). This analysis revealed a significant CdtB-dependent upregulation of the transcripts of six members of the AP-1 superfamily (Figure 1): *MAFB*, a member of the large MAFs; *MAFF*, a member of the small MAFs; *FOSB*, also known as G0/G1 switch regulatory protein 3; the Activating Transcription Factor 5 (*ATF5*); the Basic Leucine Zipper ATF-Like Transcription Factor (*BATF*); and, the JUN Dimerization Protein (*BATF3*). Among these AP-1 family members regulated by CdtB, *MAFB*, *MAFF*, and *ATF5* mRNA were found to be significantly increased with slight variations between the replicates. CdtB-induced *MAFB* upregulation could be associated with tumorigenesis [9,11], as MAFB protein exhibits oncogenic activities and promotes cancer [9]. Together, these observations suggest a possible link between CdtB and MAFB that requires confirmation.

**Figure 2:**
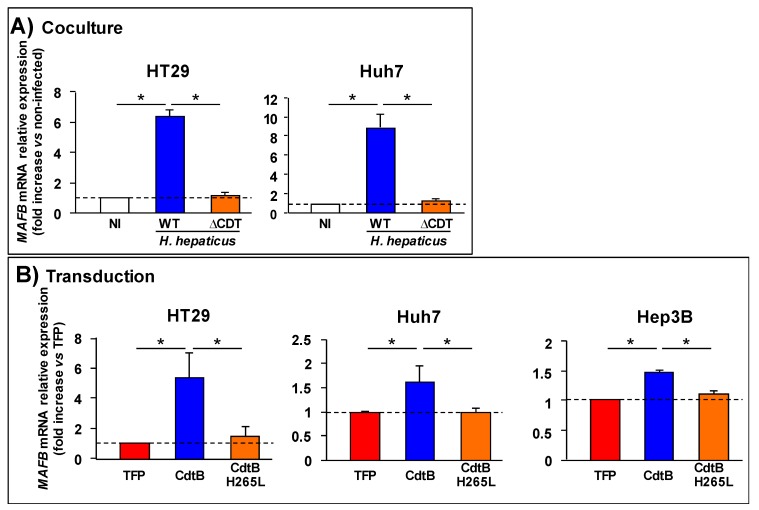
Effects of *Helicobacter hepaticus* cytolethal distending toxin on V-maf musculoaponeurotic fibrosarcoma oncogene homolog B (*MAFB*) gene expression in human epithelial cells. The expression of *MAFB* gene was determined in HT-29 intestinal cells and Huh7 hepatic cells (**A**) after a 72 h coculture with either *H. hepaticus* strain 3B1 or its corresponding isogenic *cdtB* mutant (ΔCDT) or (**B**) after a 72 h transduction with either lentiviral particles expressing the tdTomato fluorescent protein (TFP), the CdtB of *H. hepaticus* strain 3B1 and its corresponding H265L mutant CdtB (CdtB H265L) which has no catalytic activity. The level of the *MAFB* mRNA was measured by RT-qPCR and normalized relative to the reference gene, hypoxanthine phosphoribosyltransferase 1. The relative expression rate of *MAFB* gene is reported as a fold change versus non-infected control cells (for coculture experiments) or versus the TFP (for transduction experiments). The results are presented as the mean in one representative experiment (performed in triplicate) out of three. The results presented for HT-29 provided from different transduction experiments from those that are presented in Figure 1. The discontinuous line shows the basal rate in non-infected cells. * *p* < 0.05 versus non-infected cells or TFP. Abbreviations: CdtB, CdtB of *H. hepaticus* strain 3B1; CdtB H265L, *H. hepaticus* CdtB with the mutation His/Leu at residue 265 involved in catalytic activity; ΔCDT, CDT isogenic mutant of *H. hepaticus* strain 3B1; NI, non-infected; TFP, tdTomato fluorescent protein; WT, *H. hepaticus* strain 3B1 (CCUG 44777) = wild type strain.

As *H. hepaticus* colonizes the intestine and the liver, the effect of *H. hepaticus* strain 3B1 (CCUG 44777) on *MAFB* gene expression was evaluated in vitro using intestinal HT-29 and hepatocellular carcinoma (HCC)-derived Huh7 and Hep3B cell lines. A 72 h coculture, corresponding to the time that is required to observe a significant effect of the CDT [15,16] was performed. Reverse transcription with subsequent quantitative polymerase chain reaction (RT-qPCR) confirmed the microarray data, since *H. hepaticus* induced a significant increase in *MAFB* mRNA in both cell lines (HT-29 and Huh7, Figure 2A). This effect was not observed after coculture with the CDT-knock-out (ΔCDT) *H. hepaticus* strain when compared to the wild type (WT) strain. Importantly, *MAFB* mRNA level in response to *H. hepaticus* ΔCDT strain was close to the value that was determined for non-infected cells, demonstrating that CDT is the main virulence factor of *H. hepaticus* regulating *MAFB* gene expression. As expected, a significantly higher *MAFB* mRNA level was also observed in transduced hepatic (Huh7 and Hep3B) and intestinal (HT-29) cells upon the expression of *H. hepaticus* CdtB versus control TFP (Figure 2B). No significant increase in *MAFB* mRNA level was observed in response to the mutant form of *H. hepaticus* CdtB harboring a His/Leu mutation at residue 265 (H265L) that is crucial for CdtB catalytic activity [7], thus indicating that *MAFB* gene upregulation is attributed to the active CdtB. It should be noted that microarray and RT-qPCR experiments revealed that the basal level of *MAFB* mRNA is low in HT-29 cells, while its basal level is 3- and 20-fold higher in hepatic Huh7 and Hep3B cells, respectively (not shown), a result in agreement with previous data [17].

The CdtB subunit of *Helicobacter pullorum*, an emergent human foodborne pathogen implicated in several intestinal pathologies, also upregulated *MAFB* gene expression in the human intestinal epithelial cells tested (HT-29, Caco-2, and HCA7, Figure S1A).

**Figure 3:**
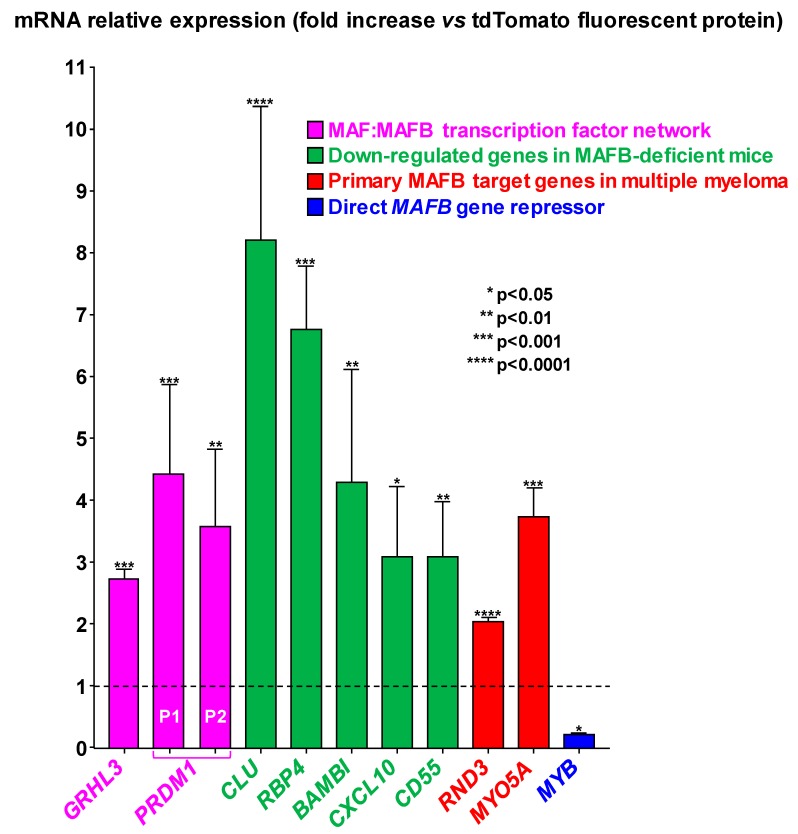
Microarray-based identification of differentially expressed MAFB target genes in response to *Helicobacter hepaticus* CdtB in intestinal epithelial cells. The expression of genes was determined in HT-29 intestinal cells using the Human GE 4x44K v2 Microarray Kit (Agilent Technologies) after a 72 h transduction with lentiviral particles expressing the CdtB of *H. hepaticus* strain 3B1 versus the tdTomato fluorescent protein (TFP). The relative expression of genes in response to CdtB is reported as a fold change versus the value for cells cultured with lentiviral particles expressing the TFP. The results are presented as the mean of four independent transduction experiments. The discontinuous line shows the basal rate in cells expressing the TFP. Asterisks denote significant results. P1 and P2 represent the probe names (P3342081 and P350451) used for *PRDM1* mRNA quantification. The data presented for *RND3* transcript are the results of 40 replicates as 10 probes for each mRNA were included on the Microarray Kit. Details are presented in Table S2 (name and sequence of the probes, the corresponding gene name, the genbank accession number, the locus and the transcript variant). Abbreviations: BAMBI, Bone Morphogenetic Protein and Activin Membrane Bound Inhibitor; CD55, CD55 Molecule, Decay Accelerating Factor for Complement (Cromer Blood Group); CLU, Clusterin; CXCL10, C-X-C Motif Chemokine Ligand 10; GRHL3, Grainyhead Like Transcription Factor 3; MYB, MYB Proto-Oncogene, Transcription Factor; MYO5A, Myosin Heavy Chain 12; PRDM1, Beta-Interferon Gene Positive-Regulatory Domain I Binding; RBP4, Retinol Binding Protein 4; RND3, Rho-Related GTP-Binding Protein RHOE; TFP, tdTomato fluorescent protein.

MAFB protein acts as a transcriptional activator or repressor, depending on the cell context and transcriptional co-factors [11]. The expression of *MAFB*-regulated genes in response to *H. hepaticus* CdtB was thus analyzed in HT-29 cells using microarray data (Figure 3). Four effectors of the MAF: MAFB transcription factor network were reported [18]. Two of them, *GRHL3* and *PRDM1*, were upregulated in response to CdtB. Among the genes that were downregulated in macrophages from MAFB-deficient mice [19], *CLU*, *RBP4*, *BAMBI*, *CD55,* and *CXCL10* were upregulated in response to CdtB. The microarray data also pointed to a significant upregulation of two primary MAFB target genes, i.e., *RND3* and *MYO5A*, which were known to be upregulated in multiple myeloma plasma cells [20].

MAFB transactivation is known to be directly repressed by Myb transcription factor (v-Myb) [21]. As expected, *MYB* gene expression was highly downregulated (five-fold decrease) in the presence of CdtB (Figure 3).

### 2.2. MAFB Oncoprotein Is Upregulated and Concentrated in the Nuclei in Response to the CdtB

MAFB localization was evaluated in vitro in response to the expression of the CdtB subunit of *H. pullorum* or *H. hepaticus* in transduction experiments. Immunofluorescence studies were performed using antibodies that were raised against MAFB protein with subsequent counting of MAFB-positive nuclei. The anti-MAFB [22] was chosen for its lack of cross-reaction with other MAFB-related proteins, such as MAFA or c-MAF.

**Figure 4:**
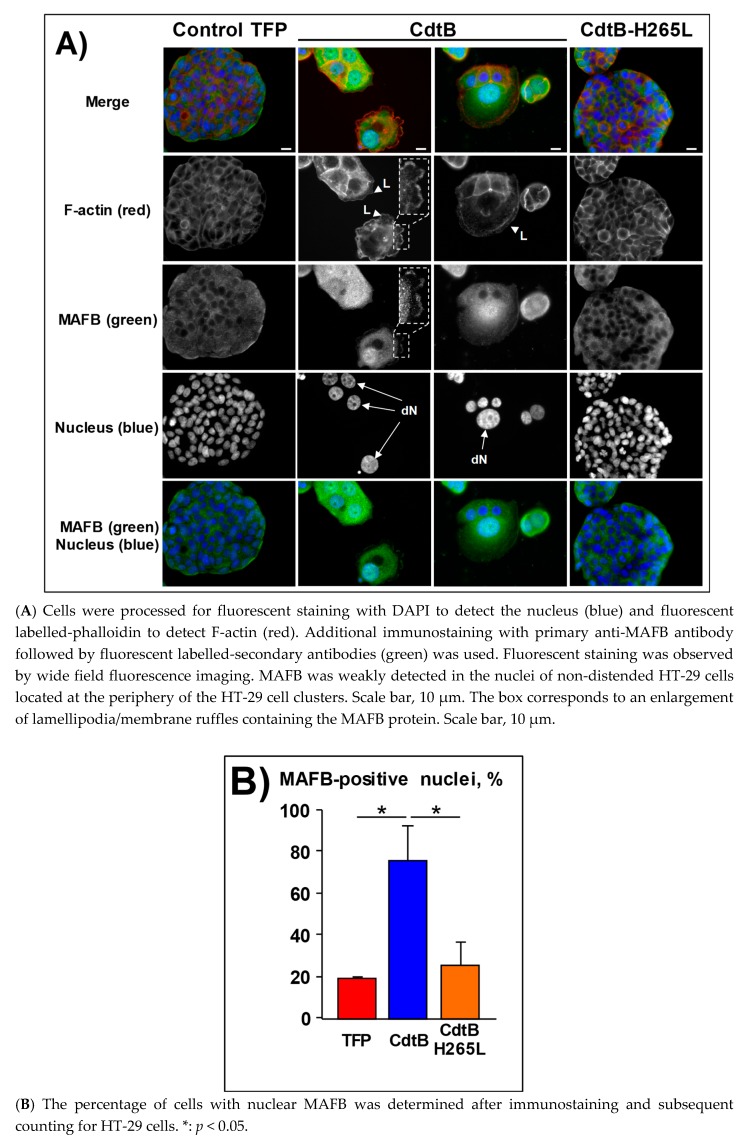

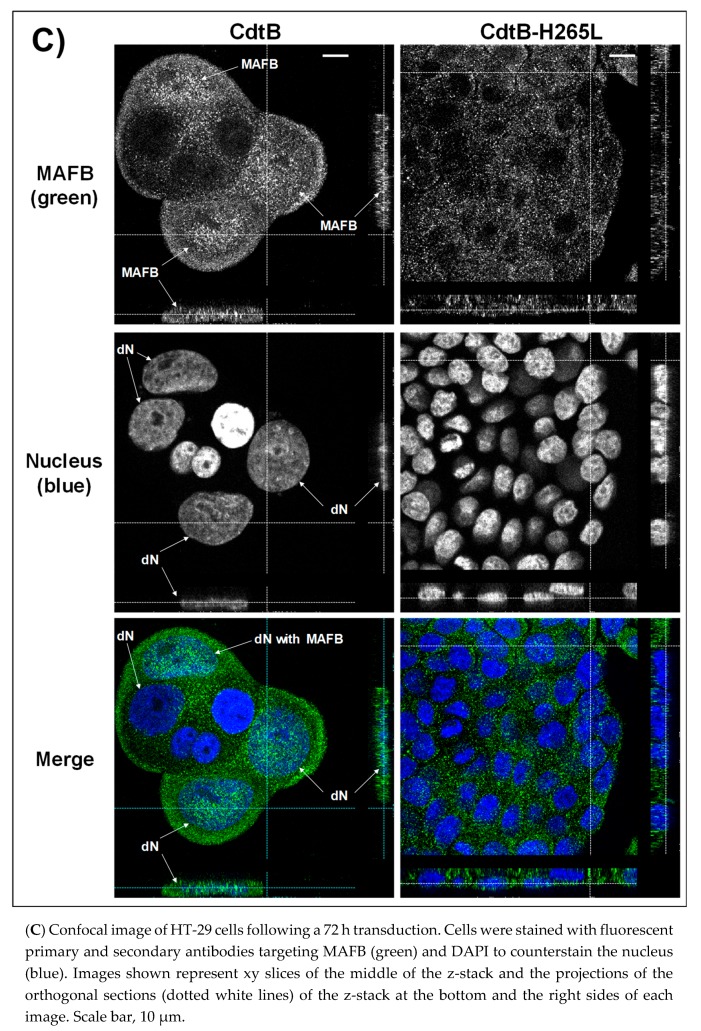
MAFB subcellular localization in intestinal epithelial cells in response to *Helicobacter hepaticus* CdtB. HT-29 cells were infected for 72 h with lentiviruses allowing the constitutive expression of the control tdTomato fluorescent protein (TFP), the CdtB of *H. hepaticus* and its corresponding mutated CdtB (H265L). Abbreviations: CdtB, CdtB of *H. hepaticus* strain 3B1; CdtB-H265L, *H. hepaticus* CdtB with H265L mutation; DAPI, 4’,6-diamidino-2-phenylindol; dN, distended nuclei; L, enlarged lamellipodia/membrane ruffles; TFP, tdTomato fluorescent protein.

Given that HT-29 cells grow tightly together, they form bunches of cells. In HT-29 control cells or expressing fluorescent proteins, MAFB was weakly detected in approximately 20% of the nuclei (Figure 4A,B). These latter MAFB-positive nuclear cells were all located at the periphery of the HT-29 proliferating cell clusters and were considered to be non-confluent proliferative cells. To the contrary, a strong nuclear localization of MAFB was observed in response to the CdtB of *H. hepaticus* (confocal analysis in Figure 4C) and *H. pullorum* (Figure S1B,C), reaching 80% in the HT-29 cells. Noticeably, MAFB also appeared to be more concentrated in the cytosol around the positive nuclei of these positive cells. Interestingly, MAFB was also detected in the periphery of large lamellipodia and membrane ruffles in some CdtB-intoxicated HT-29 cells (boxes in Figure 4A). No significant increase in MAFB nuclear content was observed in response to *H. hepaticus* H265L CdtB mutant as compared to the CdtB-induced increase in MAFB-positive nuclei, demonstrating that CdtB promotes the nuclear translocation of MAFB protein and highlighting the key role of the 265-histidine residue in the CdtB catalytic site.

**Figure 5:**
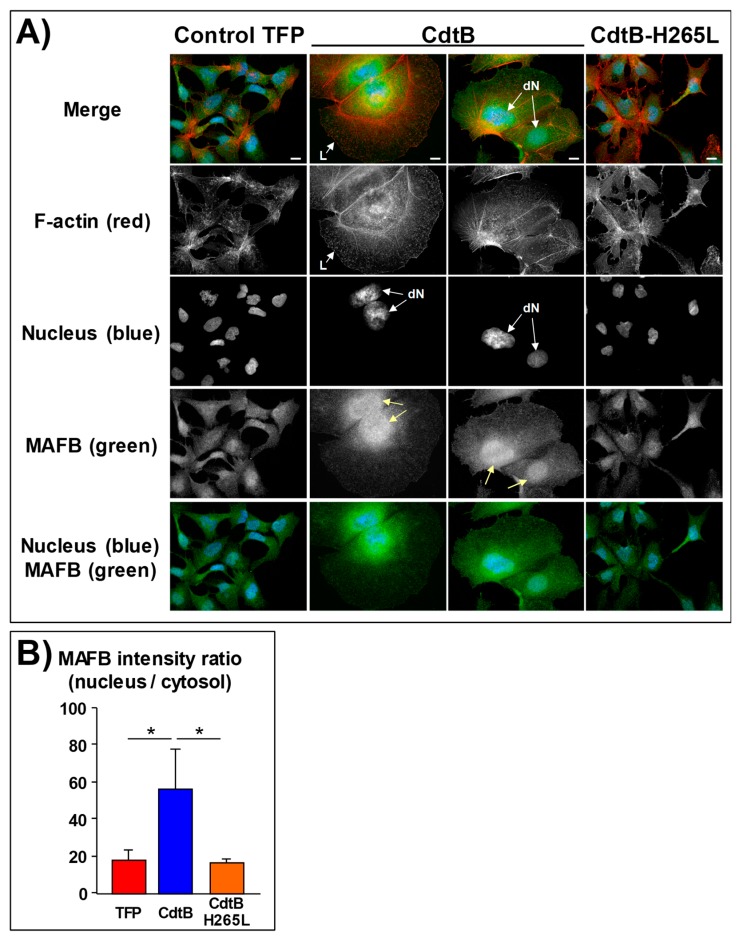
Effects of the cytolethal distending toxin of *Helicobacter hepaticus* on MAFB protein expression in human hepatic epithelial cells. (**A**) After 72 h of transduction experiments (as in Figure 4), Hep3B cells were processed for fluorescent staining with DAPI to detect the nucleus (blue) and fluorescent labelled-phalloidin to detect F-actin (red). Additional immunostaining with primary anti-MAFB antibody followed by fluorescent labelled-secondary antibodies (green) was used. Fluorescent staining was observed by wide field fluorescence imaging. Scale bar, 10 µm. (**B**) Given that MAFB is expressed in Hep3B under basal conditions, nuclear translocation of MAFB was measured after quantifying the intensity of MAFB in the nucleus versus the cytosol. For this purpose, the image processing and analysis program “ImageJ” version 1.49 [23] was used to scan and analyze black and white images of transduced cells. Nuclear MAFB was calculated according to the ratio: MAFB light intensity in the nuclei/MAFB light intensity in the cytosol. Data represent the mean of four counts in one representative experiment out of three. Arrows indicate the distended nuclei. *: *p* < 0.05. Abbreviations: CdtB, CdtB of *H. hepaticus* strain 3B1; CdtB-H265L, *H. hepaticus* CdtB with H265L mutation lacking catalytic activity; DAPI, 4’,6-diamidino-2-phenylindol; dN, distended nuclei; L, enlarged lamellipodia/membrane ruffles; TFP, tdTomato fluorescent protein.

In HCC-derived cells, the situation was different given that the MAFB protein is upregulated in HCC tissues and cells, including Huh7 and Hep3B [17]. Accordingly, Hep3B cells expressed *MAFB* mRNA and protein under basal conditions [17], resulting in the detection of MAFB protein in all cell nuclei of the control cells (Figure 5A). However, a more pronounced nuclear MAFB labeling was observed when CdtB was ectopically expressed (Figure 5A,B). MAFB protein also appeared to be highly concentrated in the cytosol around the positive nuclei of the giant CdtB-distended-positive cells presenting cortical actin-rich large lamellipodia. As expected, no increase in nuclear MAFB labeling was observed in response to the expression of the *H. hepaticus* H265L CdtB mutant.

### 2.3. MAFB Oncoprotein Is Associated with CDT-Induced Cellular Remodeling

**Figure 6:**
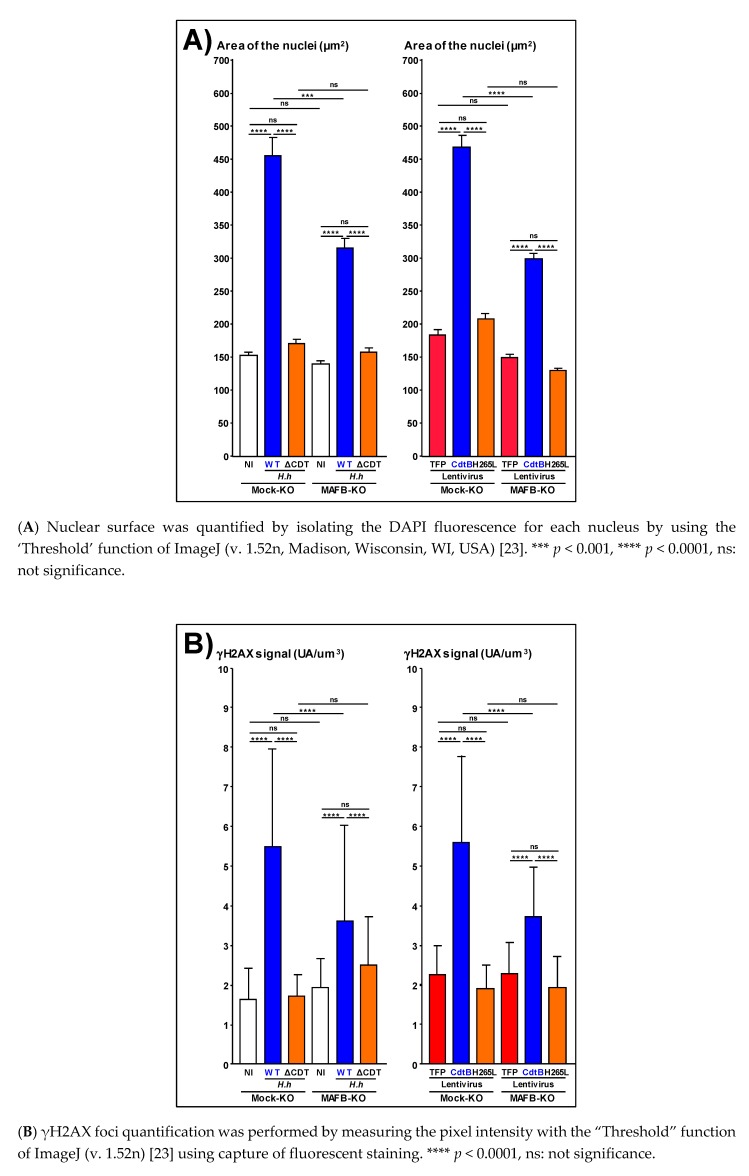

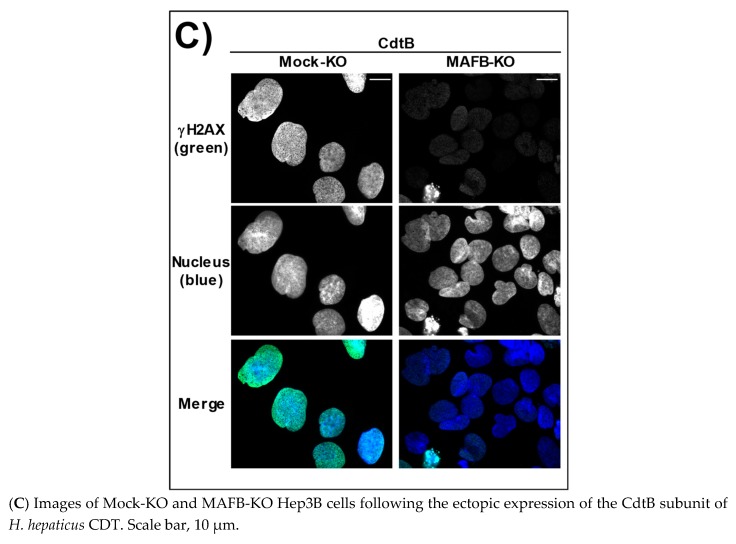
Effects of MAFB silencing on nuclear remodeling. Mock-KO and MAFB-KO Hep3B cells were infected for 72 h with *H. hepaticus* and its corresponding ΔCDT mutant strain, as well as with lentiviruses allowing the constitutive expression of the control tdTomato fluorescent protein (TFP), the CdtB of *H. hepaticus* and its corresponding mutated CdtB (H265L). These cells were processed for fluorescent staining with DAPI to detect the nucleus (blue) and primary anti-γH2AX followed by fluorescent labelled-secondary antibodies (green). Fluorescent staining was observed by wide field fluorescence imaging. The acquired images were calibrated according to the microscope software manufacturer. The results are presented as the mean in one representative experiment (performed in triplicate) out of three. A minimum of 200 nuclei were analyzed. Abbreviations: CdtB, CdtB of *H. hepaticus* strain 3B1; DAPI, 4′, 6-diamidino-2-phenylindol; ΔCDT, CDT isogenic mutant of *H. hepaticus* strain 3B1; H265L, *H. hepaticus* CdtB with the mutation His/Leu at residue 265 involved in catalytic activity; KO, knock-out; NI, non-infected; ns, non-significant, TFP, tdTomato fluorescent protein; WT, *H. hepaticus* strain 3B1 (CCUG 44777) = wild type strain.

The effects of the CDT following the MAFB extinction were analyzed with the two-way system combining the coculture and transduction experiments. We previously reported that the HT-29 cell line is resistant to the effects of the CDT during coculture experiments, but becomes susceptible to CdtB by using direct expression of the CdtB subunit in the cell during transduction experiments [5]. On the other hand, Hep3B cells constitutively express MAFB [17] and exhibit profound remodeling of the nucleus and actin cytoskeleton in response to CDT during both coculture and transduction experiments [8,15]. Thus, MAFB silencing was performed on Hep3B instead of HT-29 using the CRISPR-Cas9 technology. This genome-editing approach leads to a non-clonal cell population comprising 80% of the knock-out (KO) cells for the *MAFB* gene. Mock-KO (human crRNA negative control) and MAFB-KO Hep3B cells were subsequently infected with *H. hepaticus* and its CDT corresponding mutant strain to evaluate the effects on nuclear remodeling. No significant change in the area of the nuclei was noted between the non-infected cell lines, Mock-KO and MAFB-KO (Figure 6A,C). The size of the nuclei was significantly increased in response to the CDT, when compared to the non-infected cells or to the cells infected with the ΔCDT mutant strain, in both Mock-KO and MAFB-KO cell lines. However, a significant reduction of the increase in Hep3B nucleus size was observed in response to CDT and its active CdtB subunit in the MAFB-KO cells, as compared to the Mock-KO cells (average surface area of 315.9 μm^2^ versus 454.6 μm^2^ for CDT; average surface area of 299.6 μm^2^ versus 468.8 μm^2^ for CdtB). Similarly, a significant reduction (~1.5-fold) of the CDT- and CdtB-increase in phosphorylated H2AX (γH2AX) foci formation, a surrogate marker of double-stranded DNA breaks, was also observed in the MAFB-KO cells, when compared to the Mock-KO cells (Figure 6B,C).

Finally, MAFB silencing was also associated with the formation of narrower lamellipodia in response to the CDT (Figure S2B), as compared to Mock-KO cells (Figure S2A).

## 3. Discussion

In the present study, we demonstrated that the CdtB of *H. hepaticus* and *H. pullorum* induces *MAFB* mRNA and protein expression. Transduction and infection experiments both showed that exposure to CDT, via the CdtB subunit, promotes the expression of MAFB protein in intestinal and hepatic cell lines. MAFB was mostly found in the cytosol around the nuclei where endoplasmic reticulum localized, as well as concentrated in numerous CdtB-induced enlarged nuclei in hepatic and intestinal cells. It should be noted that cell density affects the nuclear localization of MAFB in intestinal HT-29 cells expressing TFP or CdtB-H265L. Indeed, the non-confluent proliferating cells at the periphery of (TFP- or CdtB-H265L-expressing) HT-29 cell clusters exhibited MAFB-positive nuclei in contrast to the more confluent cells in the center of cell clusters, where little MAFB nuclear location was observed. However, this was not the case in distended HT-29 cells expressing the CdtB, as they all contained MAFB in their nuclei, regardless of the cell density. Moreover, strong nuclear and perinuclear localization of MAFB was observed at the periphery of the non-confluent proliferating cell clusters in distended HT-29 intestinal cells. Cell density has already been shown to affect intracellular localization of proteins [24]. This difference between MAFB nuclear localization, depending on the cell density, might be associated with cell proliferation status and remains a matter of investigation.

MAFB also regulates some genes that are affected by CdtB [18,19,20]. As a transcription factor, MAFB might induce the regulation of these genes following its induction by the CdtB. Accordingly, the overexpression of Clusterin mRNA might be due to DNA damage that is induced by the CdtB. Indeed, Clusterin, also known as ionizing radiation-induced protein-8, is a cytoprotective chaperone that protects against genotoxic stress and suppresses DNA damage-induced cell death [25]. Thus, MAFB would be upregulated to counteract the DNA damage that is induced by the CDT. On the other hand, the decrease in *MYB* mRNA that is induced by the CdtB would occur upstream of MAFB overexpression. Indeed, MYB transcription factor is known to directly repress MAFB transactivation and maintains cell proliferation [21]. Thus, CdtB-induced MYB repression would lead to MAFB protein expression and cell cycle arrest.

MAFB was shown to be involved in actin organization in macrophages and associated with lamellipodial extensions [12]. We previously reported that Helicobacter CdtB induces cortactin upregulation and triggers the formation of cortical actin rich large lamellipodia in intestinal epithelial cells [5]. In those cells, MAFB proteins were also localized at the leading edge of the CdtB-induced large lamellipodia (Figure 4A). In hepatic cells, MAFB silencing changed the cellular response to CDT with the formation of narrower lamellipodia. Furthermore, it has been reported that *MAFB* gene expression promotes cell migration, invasion, and proliferation in vitro and tumor growth in vivo when using mouse xenograft models, in agreement with the oncogenic properties of MAFB [9,17,26]. The CdtB also alters the cytoskeleton and focal adhesion [5,14], modifying some cellular functions [13], particularly those that are linked to epithelial adherence [5]. Adherence regulates the growth, migration, proliferation, and cell death. In this context, MAFB overexpression might mediate the CdtB-dependent decreased cellular adherence. Indeed, MAFB induction by CdtB would increase *RND3* gene expression, leading to an increase in RND3 GTPase (also known as RHOE), acting as a negative regulator of cytoskeletal organization leading to loss of adherence. Moreover, RND3 plays a critical role in cell cycle arrest, cell growth inhibition, and apoptosis [27]. All of these features are hallmarks of CDT.

Besides having an oncogenic function, MAFB can play a role as a tumor suppressor-like protein [9,28], revealing a dual role for MAFB in oncogenesis. Thus, MAFB would be merely overexpressed for its tumor suppressor function to overcome the oncogenic properties of the CdtB. The relation between CdtB-induced overexpression of MAFB and these dual roles has not yet been fully elucidated, but the beneficial effect of the MAFB silencing on CDT-induced nuclear remodeling and lamellipodial extensions would be consistent with the oncogenic properties of MAFB oncoprotein. This again emphasizes the unique aspects of CDT intoxication and supports its involvement in carcinogenesis.

## 4. Materials and Methods

### 4.1. Cell Lines

The human epithelial cell lines HT-29, Caco2, and HCA7 derived from a colon adenocarcinoma, and the human epithelial cell lines Hep3B and Huh7 that were derived from a hepatocellular carcinoma were used. The cell lines were grown in their respective culture medium [5,6,15] (Invitrogen, Cergy Pontoise, France) supplemented with 10% heat-inactivated fetal calf serum (Invitrogen, Cergy Pontoise, France) at 37 °C in a 5% CO_2_ humidified atmosphere.

### 4.2. Reagents

Monoclonal mouse anti-cortactin (clone 4F-11) and monoclonal rabbit anti-phospho-histone H2AX (Ser139) (clone 20E3) were purchased from Pierce (Rockford, IL, USA) and Cell Signaling (Danvers, MA, USA), respectively. Polyclonal rabbit anti-MAFB (NB600-266) was obtained from Novus Biologicals (Littleton, CO, USA). The *MAFB* primers (F-MAFB-P3220837 CGCCAAACCGCATAGAGAAC and R-MAFB-P3220837 ACCGTAATAATAAACCCAAACAAAGAC) were designed using the Primer Express software (version 3.0.1) package (Applied Biosystems, Carlsbad, CA, USA).

### 4.3. CRISPR-Cas9 Mediated MAFB Knock-Out

SpCas9 target sequence (5′-GGTGTGTCTTCTGTTCGGTC-3′) located in the first third of the unique *MAFB* coding exon was designed using a CRISPOR algorithm [29]. Alt-R^®^-crRNA corresponding to the *MAFB* target sequence and a human crRNA negative control were purchased from Integrated DNA Technologies IDT (Coralville, IA, USA). Alt-R^®^ Hifi S.p. Cas9 Nuclease V3 (10 nM corresponding to 240 ng, IDT) was mixed with an equal molarity of each two-part gRNA (Alt-R^®^-crRNA + Alt-R^®^-tracrRNA) reconstituted following the supplier’s recommendations (IDT). Reverse transfection was performed on 40,000 Hep3B cells using Lipofectamine CRISPRMAX™ Reagent (Thermo Fisher Scientific, Bordeaux, France). At 2–3 days post transfection, a sample of cells was lysed and used as PCR template using Phire Tissue Direct PCR Master Mix (ThermoFisher Scientific). PCR amplification with subsequent Sanger sequencing of the targeted *MAFB* sequence was performed according the supplier’s recommendations with external specific primers (5′-CTCAGCACTCCGTGTAGCTC-3′ and 5′-ACGCTTGGTGATGATGGTGA-3′). Sanger data were used to quantify Indels reflecting gene knock-out (KO) with the TIDE and ICE (Inference of CRISPR Edits) algorithms [30,31].

### 4.4. Statistical Analysis for Quantification

Statistical analysis for transcriptomic analysis was performed as previously reported [6]. Other statistical analyses were performed using GraphPad Prism version 5 (GraphPad software, San Diego, CA, USA). The results are presented as the mean ± standard deviation in one representative experiment (performed in triplicate) out of three experiments. The means were compared with a non-parametric test (one-way ANOVA, multiple comparison), and the Mann-Whitney-Wilcoxon test for comparison between two groups.

### 4.5. Other Materials and Methods

The other materials and methods used in this study were previously reported. They include transcriptomic analysis using the Human GE 4x44K v2 Microarray Kit (Agilent Technologies, Les Ulis, France) with subsequent statistics [6], reagents and antibodies [5,15], cell lines and Helicobacter strains [5,6,15], construction of lentivirus plasmids with *cdtB* sequences [5,7], lentivirus production [5], coculture and transduction experiments [5,6], RT-qPCR [5,15], and immunofluorescence with subsequent image analysis (wide field and confocal imaging) [5,6,15]. The sequence of the *cdtB* of *H. hepaticus* strain 3B1 fused at its 3′ end to three repeats of the human influenza hemagglutinin (HA) epitope and that of its corresponding mutated *cdtB* sequence (A→T transversion at nucleotide 794 [5]) are available in the GenBank database under the accession numbers KT590046 and KT590047, respectively. The *cdtB* sequence of *H. pullorum* strain H495 fused at its 3′ end to three repeats of the human HA epitope is available under the GenBank accession number: JX434689.

## Supplementary Materials

The following are available online at https://www.mdpi.com/2072-6651/12/3/174/s1, Figure S1: Effects of the cytolethal distending toxin of *Helicobacter pullorum* on *MAFB* gene expression and MAFB protein subcellular localization in human epithelial cells; Figure S2: Effects of MAFB silencing on actin cytoskeleton remodeling. Table S1. AP-1 family members analyzed using whole genome microarrays. Table S2. Known MAFB-regulated genes analyzed using whole genome microarrays.
