# Supplementary material for: The Nuclear Remodeling Induced by Helicobacter Cytolethal Distending Toxin Involves MAFB Oncoprotein
Source: Toxins (Basel). 2020 Mar 12;12(3):174. doi: 10.3390/toxins12030174 (PMC7150770; doi:10.3390/toxins12030174)
Supplement: Supplementary file 1 [file toxins-12-00174-s001.pdf]

toxins

MDPI

Supplementary Materials: The Nuclear Remodeling Induced by Helicobacter Cytolethal Distending Toxin Involves MAFB Oncoprotein

Christelle Péré-Védrenne, Wencan He, Lamia Azzi-Martin, Valérie Prouzet-Mauléon, Alice Buissonnière, Bruno Cardinaud, Philippe Lehours, Francis Mégraud, Christophe F. Grosset and Armelle Ménard

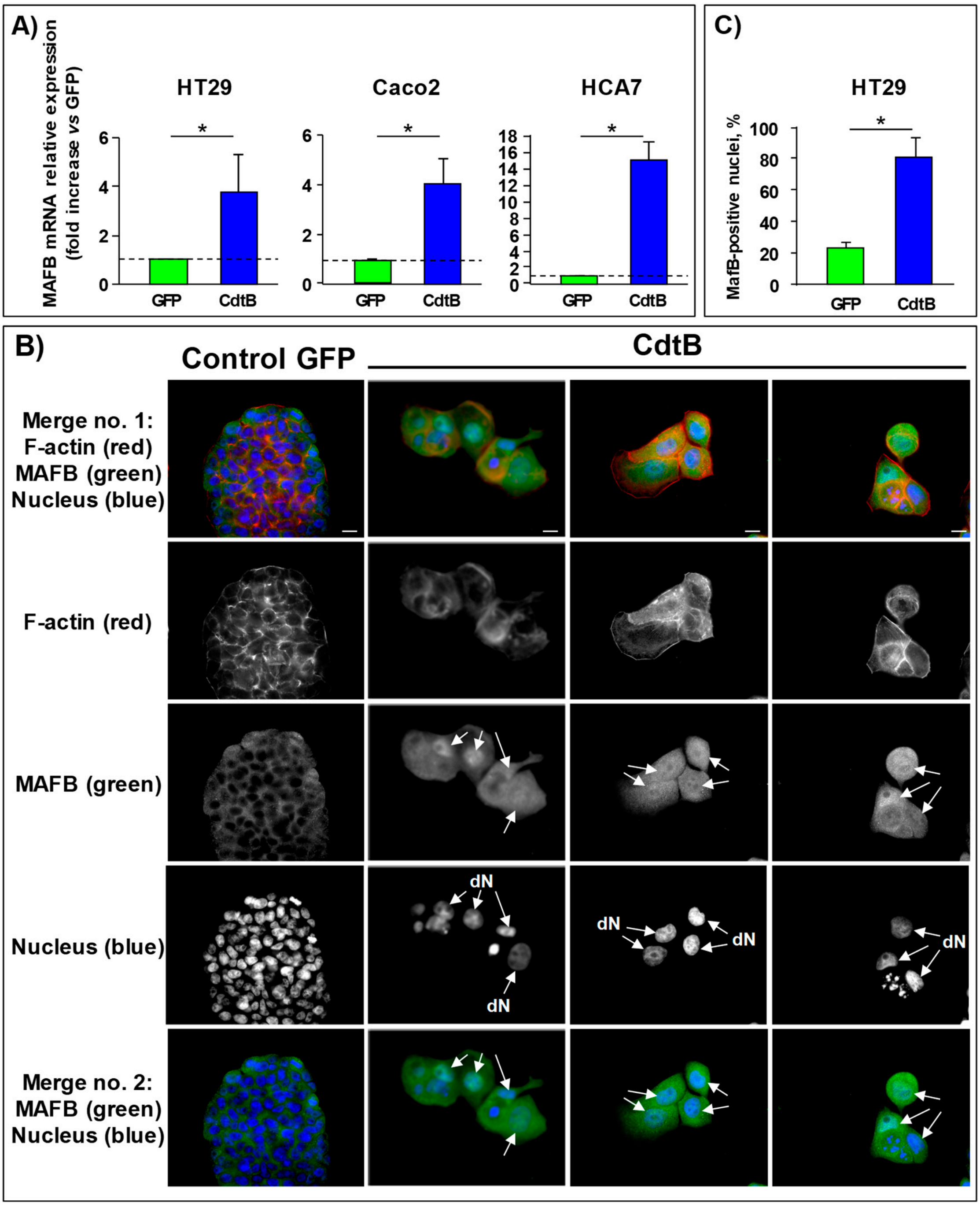

Figure S1. Effects of the cytolethal distending toxin of *Helicobacter pullorum* on *MAFB* gene expression and MAFB protein subcellular localization in human epithelial cells. **(A)** The expression of *MAFB* gene was determined in HT-29, Caco-2, and HCA7 intestinal cells after a 72 h transduction with

lentivirus particles expressing the enhanced Green Fluorescent Protein (GFP) or the CdtB subunit of *H. pullorum* strain H495 (CCUG 33840), as previously validated [5]. The expression of the *MAFB* gene was measured by RT-qPCR and normalized relative to the reference gene, hypoxanthine phosphoribosyltransferase 1. The relative expression rate of *MAFB* gene is reported as a fold change *versus* the enhanced Green Fluorescent Protein. The results are presented as the mean in one representative experiment (performed in triplicate). The discontinuous line shows the basal rate in non-infected cells. * $p < 0.05$ *versus* GFP. **(B)** After 72 h of transduction experiments, HT-29 cells were processed for fluorescent staining with DAPI to detect the nucleus (blue) and fluorescent labelled-phalloidin to detect F-actin (red). Additional immunostaining with primary anti-MAFB antibody followed by fluorescent labelled-secondary antibodies (green) was used. Fluorescent staining was observed while using wide field fluorescence imaging. MAFB was weakly detected in the nuclei of non-distended HT-29 cells located at the periphery of the HT-29 cell clusters. White arrows show the distended nuclei and MAFB in the distended nuclei. **(C)** The percentage of cells with MAFB in the nucleus was determined after immunostaining and subsequent counting for HT-29 cells. * $p < 0.05$ *versus.* GFP. Abbreviations: CdtB, CdtB of *H. pullorum* strain H495; DAPI, 4′,6-diamidino-2-phenylindol; dN, distended nuclei; GFP, enhanced Green Fluorescent Protein.

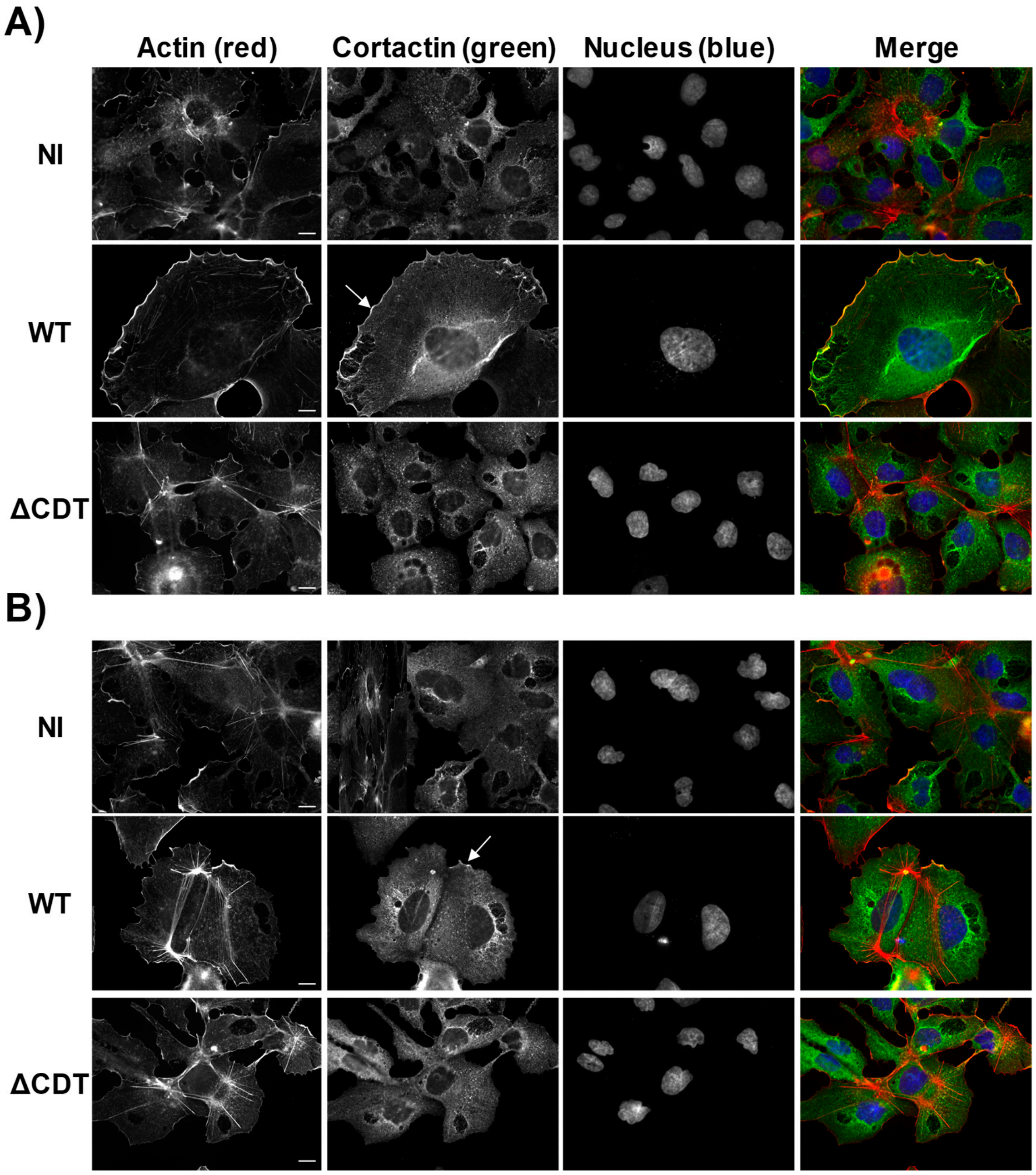

Figure S2. Effects of MAFB silencing on actin cytoskeleton remodeling. (A) Mock-KO and (B) MAFB-KO human hepatic Hep3B cells were non-infected and infected for 72 h with *H. hepaticus* and its corresponding ΔCDT mutant strain. Cells were then processed for fluorescent staining with DAPI to detect the nucleus (blue) and fluorescent labelled-phalloidin to detect F-actin (red). Additional immunostaining with primary anti-cortactin antibody followed by fluorescent labelled-secondary antibodies (green) was used [5]. Fluorescent staining was observed while using wide field fluorescence imaging. Scale bar, 10 μm. Arrows indicate cortical actin rich large lamellipodia. Abbreviations: ΔCDT, CDT isogenic mutant of *H. hepaticus* strain 3B1; DAPI, 4′, 6-diamidino-2-phenylindol; NI, non-infected; WT, *H. hepaticus* strain 3B1 (CCUG 44777) = wild type strain.
